# Perceived Safety Assessment of Interactive Motions in Human–Soft Robot Interaction

**DOI:** 10.3390/biomimetics9010058

**Published:** 2024-01-21

**Authors:** Yun Wang, Gang Wang, Weihan Ge, Jinxi Duan, Zixin Chen, Li Wen

**Affiliations:** 1 School of New Media Art and Design, Beihang University, Beijing 100191, China; 2 Academy of Arts and Design, Tsinghua University, Beijing 100084, China; wang-g23@mails.tsinghua.edu.cn; 3 The Future Laboratory, Tsinghua University, Beijing 100084, China; 4 Sino-French Engineer School, Beihang University, Beijing 100191, China; gwh-ling@buaa.edu.cn; 5 Department of Mechanical Engineering and Automation, Beihang University, Beijing 100191, China; jessieduan@buaa.edu.cn (J.D.); zixinchen@buaa.edu.cn (Z.C.)

**Keywords:** perceived safety assessment, design strategy, human–soft robot interaction

## Abstract

Soft robots, especially soft robotic hands, possess prominent potential for applications in close proximity and direct contact interaction with humans due to their softness and compliant nature. The safety perception of users during interactions with soft robots plays a crucial role in influencing trust, adaptability, and overall interaction outcomes in human–robot interaction (HRI). Although soft robots have been claimed to be safe for over a decade, research addressing the perceived safety of soft robots still needs to be undertaken. The current safety guidelines for rigid robots in HRI are unsuitable for soft robots. In this paper, we highlight the distinctive safety issues associated with soft robots and propose a framework for evaluating the perceived safety in human–soft robot interaction (HSRI). User experiments were conducted, employing a combination of quantitative and qualitative methods, to assess the perceived safety of 15 interactive motions executed by a soft humanoid robotic hand. We analyzed the characteristics of safe interactive motions, the primary factors influencing user safety assessments, and the impact of motion semantic clarity, user technical acceptance, and risk tolerance level on safety perception. Based on the analyzed characteristics, we summarize vital insights to provide valuable guidelines for designing safe, interactive motions in HSRI. The current results may pave the way for developing future soft machines that can safely interact with humans and their surroundings.

## 1. Introduction

After over a decade of development, the field of soft robotics has established a robust research foundation encompassing materials, structures, and actuation mechanisms [1]. Compared to rigid robots, soft robots exhibit unique adaptability and flexibility in various environments, and are often considered capable of providing a safe and sensible human–machine interface [2]. The above characteristics grant soft robots notable advantages in close-proximity working environments while interacting with human users, making them adept at performing diverse tasks such as medical assistance, daily life services, and social companionship. Safety has been a paramount concern in situations when rigid robots operate in spaces with a human presence, stimulating extensive research interest, particularly on collision prediction, control algorithms, and motion planning. However, these research methods and results may not be applicable to soft robots engaged in close interaction with humans.

The inherent flexibility in the structure and materials of soft robots allows them to make direct contact with users without causing harm, and the tactile interactions generated by soft robots can influence users’ feelings and behavior. Users do not merely interact with a robotic system, but coexist within the same social context as the soft robot itself [2]. In other words, when soft robots perform physical interaction with human users, the “level of safety” from a technical perspective and the “sense of safety” in the user’s actual experience may not align.

Amongst current research examining human perception of different robot types in the field of human–robot interaction (HRI), there is limited work investigating the user perception of soft robots [3]. This demonstrates a lack of comprehensive research on establishing an evaluation framework for the “sense of safety” in human–soft robot interaction (HSRI). Moreover, current work on soft robots mainly focuses on the design, fabrication, modeling, and control of soft robotic systems; there is a scarcity of studies originating from the perspective of user experience to formulate guidelines tailored for designing safe interactive motions of soft robots. Addressing these research gaps is pivotal in shifting the design approach for soft robotic product strategies from a technology-driven orientation to one driven by user experience. This transition is crucial in facilitating the effective commercialization and everyday application of soft robots.

To address these issues, this study contributes in terms of three main aspects:

- Clarifying the evaluation frameworks and methods of perceived safety in HSRI.
- Designing user experiments to assess the perceived safety of 15 interactive motions initiated by a soft humanoid robotic hand.
- Formulating insights and guidelines based on the results of user experiments for designing safe interactive motions.

## 2. Literature Review

### 2.1. Human-Hand-Inspired Soft Robot

Bio-inspired soft robots, drawing inspiration from the soft and deformable nature of biological organisms, have emerged as a transformative field in robotics. These robots are able to navigate complex environments, perform tasks with enhanced dexterity, and interact with fragile objects or living organisms without causing severe damage. The human hand serves as an excellent biomimetic model for robots, especially in tasks related to fine motor skills such as exploration, grasping, and semantic expression. Despite having similar dexterity to human hands, rigid robotic hands face significant difficulties in achieving compliance and adjusting stiffness, leading to lower adaptability [1,4]. Recent advancements in compliant materials have led to a surge in interest in soft grippers actuated by pneumatic [4,5,6,7,8,9], cable-driven [10,11,12,13,14], shape memory alloy (SMA) [15,16], and electromagnetic methods [17]. Some typical soft grippers were inspired by individual fingers, usually excluding a thumb [18,19]. Nonetheless, soft robotic hands are made to mimic the softness and adaptability of human hands as well as achieve anthropomorphic motion [20,21,22,23,24,25,26,27,28], making them a promising tactile interface for future HRI.

**Figure 1:**
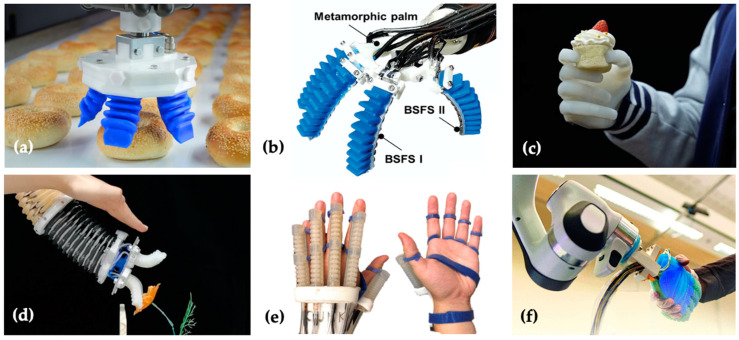
(**a**,**b**) Soft robotic gripper [29,31]; (**c**) soft neuro-prosthetic hand [28]; (**d**) human teaching soft robot to perform tasks [32]; (**e**) soft robot for hand rehabilitation [33]; (**f**) soft robotic hand with human-inspired soft palm [34]. Reprinted with permission from Ref. [28]. 2023, Guoying Gu et al.; Reprinted with permission from Ref. [31]. 2023, Wenbo Liu et al.; Adapted with permission from Ref. [32]. 2022, Wenbo Liu et al.; Reprinted with permission from Ref. [33]. 2022, Zhi Qiang Tang; Reprinted with permission from Ref. [34]. open access.

Currently, commercial soft robot grippers are mainly used for handling crops and food [29] (see Figure 1a), and have not yet been widely used in scenarios involving human contact. With the development of intelligent agents, potential applications of soft robotic hands will gradually expand towards health care, human assistance, emotional relief, and collaborative work [28,29,30,31,32,33,34] (see Figure 1b–f).

Existing research on soft robotic hands mainly focuses on their fabrication and control [1]. Considering the distinct physical and psychological gaps between human users and soft robots, which differ from the challenges with rigid robots, it has become necessary to conduct an in-depth exploration of user safety perception and assessment in HSRI.

### 2.2. Perceived Safety in HRI

Researchers have attempted to identify a comprehensive method to articulate the sense of safety. Subjective safety includes physical, social, and psychological safety, and objective safety can be measured by behavioral and environmental parameters [35]. Safety not only signifies the actual level of security, but also represents a psychological perception. In the field of HRI, safety issue has always been a key challenge, particularly in scenarios involving collaboration between humans and rigid robots [36]. Zacharaki et al. summarized a large number of related works into four categories, namely control safety, motion planning, contact prediction, and psychological factors [37]. According to the EPSRC principle [38], safety in robotics should not only include physical and material safety, but also avoid damaging the psychological, social, moral, and other important values. Whereas physical safety mainly considers physical injuries, psychological or perceived safety focuses on people’s subjective feelings when interacting with robots [39].

There is relatively less exploration of perceived safety in HRI compared to the extensive research aiming to enhance physical safety from a technological perspective. However, it is possible to obtain a widely discussed definition of perceived safety from existing work to serve as the foundational concept for this study. A large number of different terms have been used to define perceived safety [40], including sense of safety and security [39,41], psychological safety [42,43,44], and mental safety [45]. Meanwhile, words such as trust, comfort, pressure, fear, and anxiety have also been used as alternative terms for perceived safety [46]. Perceived safety can be described as not feeling fear of robots or being surprised by robots [45], or when the consequences of robot-related factors do not lead to distrust, discomfort, or lack of control over interactions [39,40,43] regarding the appearance, embodiment, motion, and social conduct of robots [44]. In this study, we adopted a generalized definition of perceived safety, referring to users’ safety-related feelings during interactions with a soft robotic hand.

### 2.3. Unique Safety Issue in HSRI

The most common failure of soft robots is actuator disfunction, which can potentially lead to safety concerns. For fluidic soft actuators, technical measures such as limited drive pressure, avoidance of bubbles and defects during manufacturing, usage of elastic covers for puncture prevention, and installation of pressure release systems can help reduce functional and structural failures. Furthermore, soft robots also have unique characteristics that introduce unprecedented danger into HSRI. Compared to the collision danger usually faced by rigid robots, soft robots pose a static danger, represented by a sudden release of elastic force [47]. Another unique danger is called “whiplash”, which refers to the phenomenon in which the tip of a whip may have a large linear velocity as a result of a small movement at the other end [47].

Furthermore, one of the most significant distinctions between interacting with a soft robotic hand and a rigid one is the potential of the former to operate in close proximity to humans and even make direct contact with its human users [47]. In the safety considerations for rigid robotic hands, the general idea is to prevent the robot from approaching humans [36,37]. Hence, the safety concerns in interactions with soft robots are not only related to their unique physical properties such as driving mechanisms or materials, but also necessitate a forward-looking consideration of their perceived safety when in close proximity to human users. Therefore, based on the unique safety concerns of soft robotic hands, this study designed related interactive motions and scenarios for assessment.

### 2.4. Existing Evaluation Frameworks

Zacharaki et al. conducted a comprehensive literature survey from 2000 to 2020, primarily focused on rigid robots, aiming to present a multidimensional safety roadmap [39]. For hazard identification, Guiochet et al. conducted a comparative analysis of safety-critical advanced robots, indicating potential hazard issues in early industrial robots and contemporary robots for human interaction [48]. For danger evaluation, Zanchettin et al. defined constraints and indicators that allowed the assessment of whether a given robot pose and velocity could be considered safe [49].

In 2019, Akalin proposed an initial framework for a perceived safety model based on human factors [39]. Rubagotti et al. proposed that factors related to the perceived safety of robots include distance, speed, approaching direction, movement fluency, predictability, size, appearance, feedback prompts, and smooth contact [46]. In 2022, Akalin et al. further identified six elements influencing perceived safety in the context of HRI, highlighting the impact of robot characteristics such as appearance (embodiment, size, shape, and posture) and motion (speed, acceleration, and proximity) [50].

Moreover, the issue of semantic and intent understanding during interaction can also impact users’ trust in soft robots and should also be considered as an essential factor in safety assessments. Akalin et al. [39] and Bartneck et al. [40] both addressed the semantic gap problem as an important measure of robots’ perceived safety and developed semantic differential scales [40].

**Figure 2:**
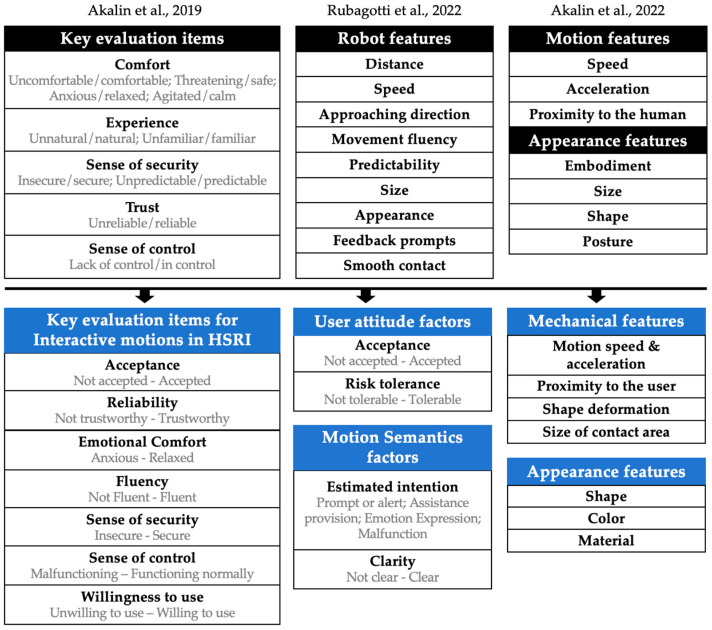
Evaluation frameworks [39,46,50].

The perspectives and categorization of the aforementioned frameworks are closely aligned with our study, serving as the primary references. As shown in Figure 2, building on these frameworks, overlapping concepts were integrated and unique characteristics of soft robots were introduced, thus crafting an evaluation framework specifically tailored to the objectives of this study. Seven key items were identified for evaluating the perceived safety of interactive motions in HSRI: acceptance, reliability, emotional comfort, fluency, sense of security, sense of control, and willingness to use. This study also explored and discussed important influencing factors, including user attitudes, motion semantics, mechanical features, and appearance features.

### 2.5. Measurement and Evaluation Methods

Commonly used measurement and evaluation methods of perceived safety include questionnaire surveys, physiological measurements, behavioral assessments, and direct input devices [40,44,46]. As shown in Table 1, several questionnaires have been used to evaluate the subjective feelings of safety of users towards robots, including the Godspeed Series Questionnaire (GSQ) [40], Psychological Scale for Safety of Humanoid [42], Negative Attitudes toward Robots (NARS) [51,52], and Robot Social Attributes Scale (RoSAS) [53].

In order to selectively present situations for evaluation, rapidly collect data, and pre-vent accidental harm, video assessment is an effective, efficient, and flexible experimental method used to assess perceived safety. Akalin et al. conducted a series of detailed studies on human safety feelings and developed a questionnaire for evaluating users’ sense of safety and security towards HRI videos [39].

Physiological measurements for perceived safety assessment usually encompass heart rate, galvanic skin response, electromyography, gaze, and respiration [45,54,55]. However, the interpretation of physiological signals may be subject to bias due to the subjective processing by different researchers.

Several researchers suggested that integrating quantitative and qualitative measurements is essential for establishing a reliable assessment of HRI [44,54]. This study incorporates multiple measurement methods, including questionnaire surveys, behavioral measurements (gaze analysis), and self-report (user interview).

## 3. Materials and Methods

To investigate the perceived safety in HSRI, this study employed the soft robotic hand as the main interacting subject for user experiments. Based on the mechanical characteristics and unique safety issues of the soft robotic hand mentioned in previous sections, along with common social touch behaviors observed in daily life, a total of 15 interactive motions (see detailed descriptions in Section 3.2) executed by a soft robotic hand on the human forearm were selected as the primary experimental stimuli. To protect participants’ physical well-being, minimize psychological pressure, and mitigate influence of unrelated variables, the 15 motions were presented to participants in video format for subsequent questionnaire-based assessments of perceived safety. Additionally, participants’ visual attention features during the experimental process were captured using a remote eye-tracking device (Eyeso Ec50).

The experimental design and data analysis were structured around four key research questions:

- RQ1. During direct or close contact with users, which interactive motions of the soft robotic hand are perceived as safe?
- RQ2. What semantics and mechanical characteristics are implicated in participants’ perception of safe interactive motions?
- RQ3. Do users’ acceptance and risk tolerance of the soft robotic system influence their assessments of perceived safety during interactions?
- RQ4. During the interaction, what specific components of the soft robotic hands serve as the primary visual focal points for users’ assessments of perceived safety?

### 3.1. Soft Robot Hand

**Figure 3:**
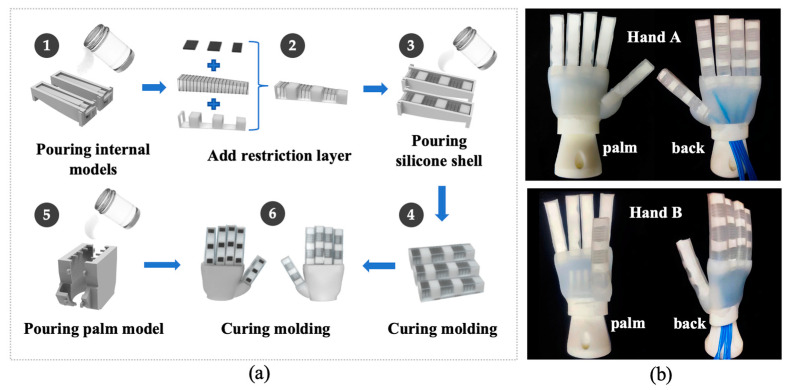
(**a**) Fabrication process of the soft robotic hands; (**b**) final result.

As shown in Figure 3a, the finger consists of a flexible actuator made of elastic material (Ecoflex 00-10, Smooth-On, San Francisco, CA, USA) and an external limiting layer. The non-stretchable Kevlar fiber wrapped around the actuator limits its radial expansion. Finger-like joint bending was achieved by adding a carbon fiber board and wrapping heat shrink tubing on the outer layer. The elastic shell of the fingers on the outside was made of elastic material (Ecoflex 00-10). The palm was also made of elastic material (Dragon Skin 10 MEDIUM, Smooth-On, USA), which connected the base of the fingers to the palm through silicone. Two hands with different thumb orientations were prepared for user experiments (see Figure 3b).

### 3.2. Interactive Motions

To mitigate experimental biases arising from an excess of variables, the interactive motions involved in this experiment primarily encompass robot-initiated touch. Touch experiences hold significant importance for humans, serving as a continuous, often unconscious way of organizing our social experiences [2]. Subsequent to the robot-initiated touch, the social attributes of the robot in interaction with humans become more pronounced. Mazursky et al. identified two distinct natural touches by robot caregivers in medical settings, which are Functional Touch involving executing physical tasks, and Emotional Touch focusing on providing comfort and soothing [56].

In HRI, even when robot instructions are clear, individuals who are approached or touched by the robot might not fully comprehend the robot’s intent, especially if they are not the originators of the instructions. Consequently, this experiment aimed to explore users’ intuitive perception of safety during touch motions initiated by the soft robotic hand, or motions resembling touch, without specifying a particular working environment for the soft robotic hand. Thus, the motions themselves became the subject for evaluation.

**Figure 4:**
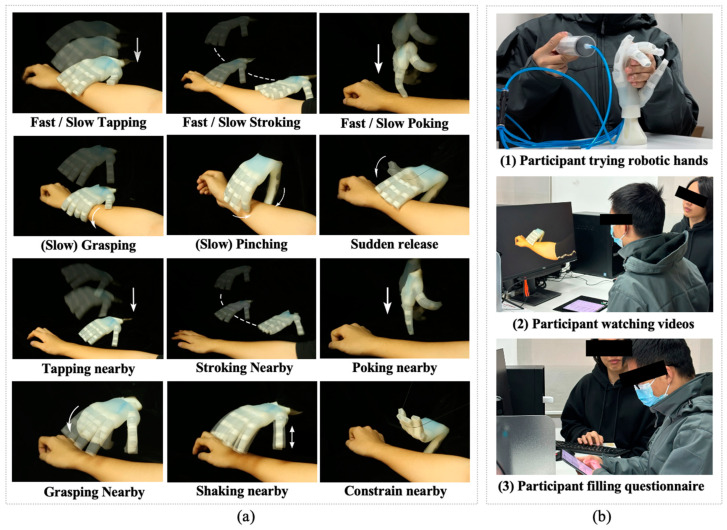
(**a**) Recorded interactive motions; (**b**) different stage of user experiment.

Five of the most common touch motions, namely tapping, stroking, grasping, poking, and pinching, were selected as primary experimental stimuli (see Figure 4a, Table A1 and Videos S1–S15 in the supplementary materials). The variables for these motions include varying speeds and proximity to the user. Additionally, because the soft robot presents two specific risk scenarios that are not encountered by rigid robots (refer to Section 2.3), three specific motions, namely sudden release, constrain nearby, and shaking nearby, were included in the experimental stimuli.

To execute the motions, the soft hand was installed on an AUBO-i5 robotic arm (AUBO-ROBOTICS, China) and controlled through online programming via teaching. Finger air pressure was regulated by a pneumatic system, with bending air pressure maintained between 31 and 35 KPa. The speed of the motions was achieved by controlling the joint movement speed of the robotic arm. Due to the safety design of the robotic arm, its maximum joint rotational speed did not exceed 148.74 °/s. Consequently, the movement speed of the action sequences was controlled within 10% to 100% of the robotic arm’s maximum joint speed, and was adjustable according to the actual motion requirements. To ensure higher naturalness and smoothness of the designated interactive motions, the collaborative control adjusted by the researchers between the robotic arm and the pneumatic system enabled the soft hand to complete the motions. The contact forces of all the motion sequences, tested using a flexible pressure sensor (CNSCAN CN5101), were found to be lower than 0.1 N.

### 3.3. Perceived Safety Assessment Questionnaire

As mentioned in Section 2.4, Akalin’s 2019 perceived safety assessment questionnaire [39] served as the primary reference for questionnaire design in this study. Given the experiment’s focus on relatively brief interactive motions, detailed emotion evaluation questions were simplified. Additionally, since the soft robotic hand has not been used as a complete product for experiences, technical acceptance questions were also simplified. The final experiment employed a 9-item 5-point scale questionnaire, covering acceptance, reliability, emotional comfort (relaxation/anxiety), fluency, sense of security, sense of control, willingness to use, motion semantics, and general safety. At the end of the questionnaire, a separate ranking question was included for participants to prioritize different physical factors of the soft robotic hands as their evaluation criteria, such as the motion speed, its position relative to the human forearm, the level of the deformation upon contact, the motion semantics, and the size of the contact area. All the scores were further calculated based on the entropy weighting coefficients of the first seven dimensions, and they were cross-validated with relevant questionnaire items (see the complete questionnaire in Table A3).

### 3.4. Participant Recuitement and Catagorization

Participants were recruited online as safety assessors for the soft robotic hand, and required to fill out a pre-test questionnaire for qualification review, answering a series of questions encompassing demographic information, educational background, attitude, acceptance levels of robots and soft robots, the importance placed on safety issues, and willingness to tolerate risks during interactions with soft robots.

A total of 33 individuals took part in the experiment. Following the removal of inconsistent data, the final analysis was performed using the data from 30 participants (12 males, 18 females, aged 18–31, M = 21.63, SD = 5.37, see Table A2). Based on their acceptance and risk tolerance of soft robots in the pre-test questionnaire, participants were categorized into the high acceptance group (HA, *n* = 16) or moderate acceptance group (MA, *n* = 14), and the high-to-moderate risk tolerance group (HMT, *n* = 18) or low risk tolerance group (LT, *n* = 12). Consequently, 3 types of participants were identified, namely cautious (MA/LT, *n* = 8), conservative (MA/MT, *n* = 6), and proactive (HA/HMT, *n* = 12). It is worth noting that only 4 participants were catagorized as the HA/LT type, all of whom majored in mechanical engineering or biomedicine. Due to their advanced understanding of robotic principles and risks, they exhibited a high acceptance level and stringent safety expectations of soft robotic technology. This type was therefore named “tech-savvy”, to some extent reflecting an expert perspective.

### 3.5. Experiment Process

Qualified participants were invited to a dedicated laboratory, and requested to voluntarily sign an informed consent form. Their IDs and overall condition upon entering the lab were recorded by experimenters.

The experimental environment was quiet, with moderate temperatures, and devoid of unrelated personnel. As shown in Figure 4b, participants initially had a free interaction with the soft robotic hand in the experience area for 2 min, which was not connected to the robotic arm and could be inflated using a syringe to simulate activation. Experimenters guided participants to explore several typical interactive motions that would later appear in the experiments. After the experience, participants were informed that their role in the experiment was that of a safety assessor, and that they would watch a total of 16 videos (1 test video and 15 formal videos) of interactive motions initiated by the same soft robotic hand that they just experienced, and then complete a safety assessment questionnaire. The overall time required was 15–20 min.

Experimenters guided the participants to the experimental area, explained the operation interface, confirmed the participants’ understanding of the process, calibrated the remote eye-tracking device, and initiated the experiment when the system and participants were ready. Participants began watching experimental videos, each lasting 10 s and playing only once. After each video, the screen went black, and participants filled out the assessment questionnaire on a mobile device. Upon completion, with the participant’s consent, the next video was played.

The first video viewed by each participant was a test video featuring an unrelated motion, during which participants could ask experimenters about unclear aspects of the questionnaire. Following the test video, participants watched the formal videos in a consistent order. Experimenters only asked participants if they were ready for the next segment and refrained from further intervention.

After completing all experiments, participants were invited to another room for an interview lasting 10 to 15 min conducted by another experimenter. Each participant received 30 Chinese Yuan and a small gift as compensation. Throughout the entire experiment and data processing process, the privacy information of all participants was treated with confidentiality.

## 4. Results

### 4.1. Experiment Results

#### 4.1.1. Characteristics of Motions Perceived as Safe

**Figure 5:**
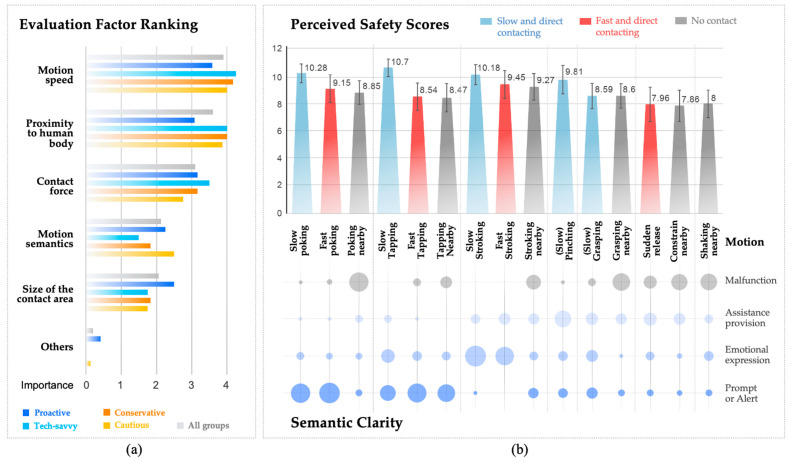
(**a**) Ranking of evaluation factors based on self-report of participants from different groups; (**b**) perceived safety score and semantic clarity of all the 15 interactive motions.

As shown in Figure 5a, based on user self-reports regarding the ranking of evaluation factors, the motion speed of the soft robotic hand and its proximity to the human forearm emerged as the two most critical factors influencing their perceived safety assessment. These were followed by the contact force of the soft robotic hand (deformation level), the motion semantics (estimated meaning of the motion), and the size of the contact area (single or multiple robotic fingers). This result aligned with the overall trend in perceived safety ratings. From the perspectives of motion speed and proximity, we can categorize the 15 interactive motions into three major groups, which are (1) slow and direct contact; (2) fast and direct contact; and (3) no contact. The complete result of the paired t-tests examining score differences across all motions is listed in Table A4. It can be observed that the slow motion group exhibits the highest level of significance.

As shown in Figure 5b, among the 15 interactive motions, slow and direct contacting motions tended to have higher perceived safety scores. As the motion speed increases, the perceived safety decreased. Motions that are considered to have certain social behaviors and involve direct contact with users are often perceived as safer (slow poking, slow tapping, and slow stroking), while motions near the user’s forearm without actual contact were most likely perceived as faulty (except for tapping), resulting in decreased perceived safety scores. The perceived safety scores between fast actions and medium-speed non-contact actions are mostly not significant.

Furthermore, simple contact motions such as poking, tapping, and stroking had relatively higher perceived safety scores, while motions involving more complex deformations, such as grasping and pinching, showed a significant decrease in perceived safety (*p* < 0.01). For actions like sudden release, constrain nearby, and shaking, which differ from common interpersonal contact actions and exhibited more resemblance of mechanical faults, their perceived safety was significantly lower than other motions (*p* < 0.01).

#### 4.1.2. Semantic Clarity

Based on the evaluation of factor rankings, when the motion aligned with users’ safety requirements in terms of speed, position, and force, the clarity of motion semantics, meaning users’ ability to clearly interpret the meaning of the motions, would impact the overall perceived safety. In the questionnaire, four typical tactile behavioral semantics were provided for participants to choose from: prompt or alert, emotional expression, assistance provision, and malfunction. As shown in Figure 5a, A four-dimensional vector was constructed to assess the semantic clarity of each motion, determining the participants’ positive or negative semantic inclination, with malfunction defined as negative semantics. We conducted a correlation analysis between the perceived safety scores and the positive or negative semantic clarity. The results demonstrated that when the negative semantic clarity of interactive actions increased, there was a significant decrease in perceived safety (r = −0.661, *p* < 0.01). However, when the motion semantics was not sufficiently clear, whether positive or negative, it did not significantly affect users’ perceived safety.

#### 4.1.3. Differences in Participant Acceptance and Risk Tolerance

**Figure 6:**
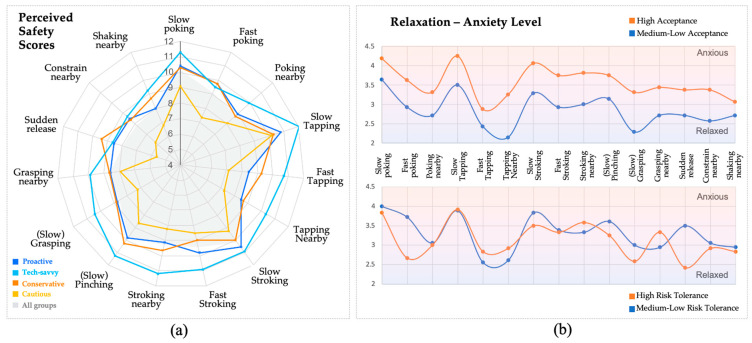
(**a**) Different perceived safety scores between the four different participant types, compared to average scores; (**b**) trends in level of relaxation between participants with different levels of soft robot acceptance and risk toleration.

Through data analysis using SPSS, the perceived safety scores of proactive, tech-savvy, conservative, and cautious participants all conformed to a normal distribution. As shown in Table 2 and Table 3, statistical results showed significant variations in perceived safety ratings across all participant groups, and most participant types, with the exception of the comparison between proactive and conservative types. As shown in Figure 6a, it is notable that the cautious group consistently rated lower scores for each motion compared to the other groups, particularly in the instances of sudden release and fast poking.

Moreover, participants with different acceptance levels and risk tolerances showed a consistent overall scoring trend (acceptance: r = 0.961, *p* < 0.01; risk tolerance: r = 0.809, *p* < 0.01). It can be found that HA participants’ perceived safety scores were significantly higher than those of MA (t = −15.911, *p* < 0.01), and LT participants’ perceived safety scores were significantly lower than those of HMT (t = 3.485, *p* < 0.01). Except for fluency and assessment of system status, other sub-items generally followed the patterns described above. Figure 6b illustrates the rating trends of participants from different groups on the relaxation–anxiety sub-item, which was the one most strongly associated with emotional responses in safety assessment. The differences among the four participant types also align with these patterns.

Gender differences in perceived safety scores were also investigated. Firstly, we used the Shapiro–Wilk test on small sample sizes to verify the normal distribution of pre-questionnaire acceptance scores, pre-questionnaire risk sensitivity scores, and perceived safety scores for all 15 motions. The results indicated that the data were generally normally distributed. Subsequently, t-tests were conducted to compare the perceived safety scores of male and female participants. Significant differences were found only in Fast and Slow Tapping (*p* < 0.05). While the data suggested that female participants generally gave lower perceived safety scores than males, most differences were statistically non-significant.

#### 4.1.4. Gaze Features during Interaction

**Figure 7:**
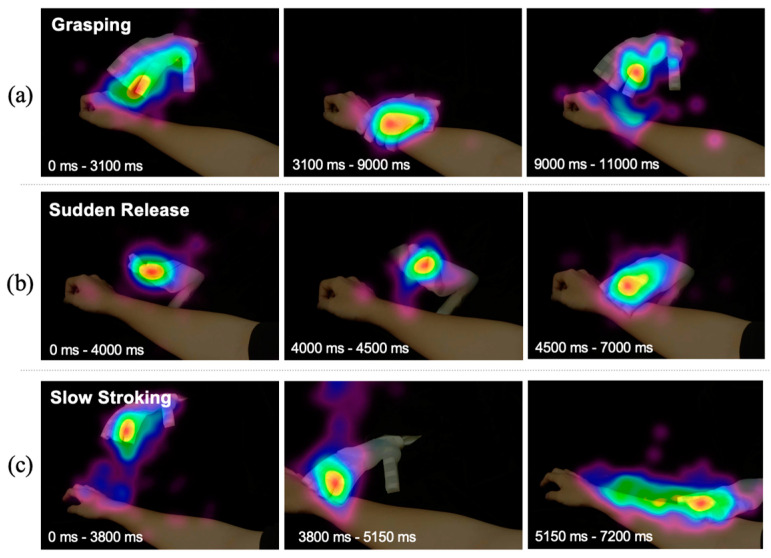
Gaze heatmaps in different motion stages of (**a**) grasping, (**b**) sudden release, and (**c**) slow stroking.

A remote eye-tracking system (Eyeso Ec50) was utilized to capture and analyze the gaze data of users watching the experimental videos, examining primary gaze points and changes in gaze trajectories through eye movement trajectories, heatmaps, and other methods. It is evident that throughout the entire experiment, participants’ gazes remained focused on the soft robotic hand in the video, ensuring that the motions and appearance of the soft robotic hand were the central stimuli in the experiment. As shown in Figure 7a–c, based on the motion status of the soft robotic hand, the videos were divided into three stages: (1) pre-motion: both the soft robotic hand and the human forearm remained stationary, participants’ primary gaze point was at the fingertips of the soft robotic hand; (2) during motion: the participants’ gaze shifted from the fingertips to the contact point between the soft robotic hand and the human forearm, and followed the entire motion of the soft robotic hand without a specific focal point, as shown in Figure 7b; (3) post-motion: participants’ gaze concentrated again on the fingertips of the soft robotic hand and gradually dispersed to the edges of the screen, representing their exploration of the surrounding environment in the video after the motion was completed.

### 4.2. Interview Findings

Based on grounded theory [57], the experimenters organized and conducted multi-level coding on the transcripts of interviews with 30 participants, based on a corpus comprising 300 min of voice recordings and transcribed textual data totaling 22,505 Chinese characters. The findings can be categorized into the following three main themes.

#### 4.2.1. User Interpretations of Motion Semantics

When people are familiar with the situation caused by robot motion, they tend to feel confident and safe about the actions made by robots [39]. Most participants judged motion semantics based on personal experience, imagining real-life scenarios. Many believed that the difficulty in determining the intention of motions would affect their perceived safety of the soft robotic hand. P22 mentioned, “*I would think stroking is for some medical or therapeutic uses, like massage*”. The motions in close proximity led to a certain level of caution. P35 noted, “*I might feel a bit scared, suspecting if it’s a malfunction or if it might suddenly strike*”. Faced with unknown motion sequence, participants generally exhibited alertness, attempting to predict its purpose. If predictions failed, trust in the soft robotic hand could decrease. P4 mentioned, “*Being in this unknown state feels very unsafe*”. P18 added, “*If an action goes beyond my expectations, I would be afraid of its next move*”. In the ranking of evaluation factors, the motion semantics ranked lower, possibly related to participants perceiving it as corresponding to actual danger. As mentioned by P17, “*Semantic deviation may have an impact on psychological or mental aspects, but the others factors may actually cause some physical harm*”. A few participants emphasized the importance of clear semantics and the relationship between semantics and mechanical parameters. P33 said, “*I think, under the premise of safety, what it expresses is more important*”. P34 believed, “*Semantics is a dominant factor; it can be said that it determines other mechanical parameters*”.

#### 4.2.2. Attitudes towards Mechanical Parameters of Interactive Motions

Based on the frequency of keywords mentioned by participants, it can be observed that they were most concerned about the motion speed, which was consistent with the actual ranking of evaluation factors (see Section 4.1.1). (1) Motion speed: fast motions exhibited phenomena such as rapid starts, abrupt stops, and short durations, leading participants to associate them with difficulty in reacting promptly, difficulty in control, and the generation of greater force. This resulted in participants feeling alert, startled, and uneasy, being hard to accept, suspecting faults, and “*wanting to avoid*”. Slower motions were more easily accepted and considered as more reliable, safe, and comfortable, even if their efficiency might be compromised. (2) Contact force: participants mentioned that with minimal deformation, the contact force was usually small, and the motion was gentle, interesting, and cute. P10 mentioned, “*A pinch, like the action of a small animal*”, and P25 noted, “*Robot’s Finger deformation proves that it’s quite soft, so the potential harm to the human body may be relatively small*”. Stronger force may lead to more noticeable functionality. P23 expressed, “*If it’s gentle, I think it’s reminding me. If it’s heavy, it might be warning me*”. (3) Proximity: some participants had varying responses based on the approaching angle of the robotic hand. Participant 19 pointed out, “*The issue of touching the arm, sometimes it’s vertical, sometimes it’s parallel, giving me two different feelings*”. When the soft robotic hand was close but not in direct contact, participants felt anxious and uneasy, suspecting faults and fearing sudden attacks. (4) Size of contact area: for motions with a small contact area, such as fingertip tapping, some participants found them in line with human movement patterns, acceptable, and safe. Motions involving the entire palm’s contact (stroking especially) were perceived by some as resembling human communication, conveying emotions. However, some participants also found them too offensive, strange, and somewhat threatening, resembling petting or bathing.

#### 4.2.3. Attitudes towards Product Visual and Tactile Features

Researchers asked questions in terms of three aspects of the soft robotic hand, namely shape, color, and texture, to understand which product features participants believed would impact their sense of safety. Among the 13 participants (43% of the total number) who explicitly expressed a preference for anthropomorphism, 83.3% belonged to the high acceptance group, while 75% opposing anthropomorphism were categorized in the moderate acceptance group. (1) Shape: form can evoke intuitive feelings, and a strange structure may cause discomfort. P19 suggested, “*The size of the hand can be between that of a child and an adult, which would give me a safer feeling*”, and P20 stated, “*Its shape should be based on the application scenario it serves*”. (2) Color: Several participants highlighted the advantages of transparent materials, as they could showcase internal structures, confirm the absence of rigid components, and enhance trust in safety. P23 mentioned, “*Transparent materials allow me to easily observe its internal conditions*”. However, for participants less familiar with robots, complex internal structures might induce a psychological burden. P15 noted, “*Just like I don’t need to pay attention to what’s inside a computer, it would make me feel complex or messy*”. Regarding specific colors for the material, participants preferred light or pure white colors of industrial materials. P4 suggested, “*Avoid very alarming, eye-catching colors*”, and P2 mentioned, “*Keep the color simple, not too flashy*”. (3) Material: Most participants found the current silicone texture satisfactory, with a moderate softness and hardness, but slightly sticky and prone to collecting lint, which might be considered unhygienic. Some participants suggested that the soft robotic hand could be smoother, drier, and flatter, but not excessively smooth, retaining some stickiness. P7 noted, “*Don’t give people a very oily feeling*”. In scenarios involving contact with the human body, participants suggested trying textures including woven fabrics and leather, and expressed anticipation for such variations.

## 5. Discussions

Based on the experiment results and interview findings, the following key insights can be recapitulated to provide valuable guidelines for the development of safe interactive motions with soft robotic hands.

- Approaching and touching users at a slow, acceptable pace. Although the soft robotic hand is not likely to cause significant physical harm to users, the speed of approach and contact motions remains the most important factor influencing user perceptions of safety. When designing close interaction tasks for the soft robotic hand, it is important to establish a safe proximity and drive the hand at slower speeds within this safe range. When the soft robotic hand makes direct contact with users, variations in motion speed can convey different emotions, with faster actions leaning toward alerting and slower actions tending to express emotions.
- Ensuring the perceptibility of mechanical safety features. In the interaction between the soft robotic hand and the user, it is essential to enable users to clearly perceive the inherent softness of the material and the gentle nature of potential contacts. Prior to actual contact, the use of materials with a certain degree of transparency can visually showcase the palm or joints without rigid mechanical components, fostering user trust in the flexible structure of the robotic hand. During actual contact, the deformation of materials that closely match the bending of human hands helps users better understand the force exerted by the soft robotic hand through both visual and tactile cues.
- Informing users about the interaction purpose prior to physical contact. After users have developed trust in the mechanical performance of the soft robot, their expectations regarding interaction purpose will significantly influence their safety judgments during actual interactions. Motions that are difficult to comprehend, deviate from prior expectations, or are unpredictable in their subsequent developments can erode user trust in the system, leading to alertness and apprehension. The robot should clearly inform users about the purpose and steps of the intended motion before the interaction, especially for new interactive motions. If a motion might alter the user’s state (e.g., assisting the user in standing up), users should be given ample time to establish psychological preparation, and their consent should be obtained before motion execution. In emergency situations, clear and prominent alerts should be used to provide users with explicit warnings.
- Avoiding overly complex motion sequences. Simplified motions can alleviate user anxiety, especially for individuals with limited technology acceptance. If interaction tasks involve multiple complex motions, it is suggested to break them down into simple and predictable movements, incorporating time intervals in between.
- Providing clear demonstration of system status when near a user. When the soft robotic hand encounters obstruction or actuation failure, it is crucial to provide users with timely cues through visual, auditory, or other means to ensure their clear understanding of the current system status. In situations where the soft robotic hand cannot move to a safe area, users should be promptly alerted to evacuate and maintain a safe distance.
- Minimizing the frequency and the time duration of direct touch. Although most users, upon understanding the features of the soft robotic hand, might be inclined to accept close proximity interaction, it is advisable to minimize the frequency and duration of direct touch. Frequent and prolonged contact may lead to sensory desensitization, and sudden changes in motion states during such desensitization could potentially startle users.
- Ensuring user control at all times. Errors in the interaction system are unavoidable, and users should be given sufficient control to enable them to deactivate the robot at any time in actual interactions. Emergency control through physical buttons is necessary, and these buttons should be located in positions that are easily accessible to users but not prone to accidental activation.

There are several limitations in this study. In terms of the mechanical capabilities of the soft robotic hand, our experiment apparatus comprised a system incorporating both soft and rigid robots, and the pneumatic-driven soft robotic hand exhibited a highly restricted force generation capacity (<0.1 N), in contrast to the human finger, which can generate roughly 30 lb per square inch during bending. Given the mechanical characteristics of the soft robotic hand, pneumatic-driven movements were inherently slow compared to those of the rigid robot arm, resulting in a combined motion profile. Morphologically speaking, the bending of the soft robotic hand represented a continuous change, whereas the human hand features joints, and the material of the soft robotic hand differed markedly from human skin. All the above factors may have influenced user experience and safety assessment during the experiment.

In terms of conducting group analysis on the participants, there was a notable discrepancy in the number of individuals across the four participant types, ranging from a maximum of 12 to a minimum of 4. With smaller sample sizes within each type, the comparative analysis of data may have certain limitations. Furthermore, we observed a significantly higher safety rating in the tech-savvy group (HA/LT), which might relate to their advanced understanding of robot failure. When they observed minimal physical harm caused by the soft robotic hand during the experiment, they tended to give consistently higher ratings for the majority of motions (see Figure 6a), potentially introducing a certain level of bias.

In terms of motion semantics assessment, a minority of participants reported that for certain motions, they found it challenging to make only one choice among the options. They believed that motions of the soft robotic hand might have multiple meanings but ultimately chose one. In real-world applications, interactive motions of soft robots may simultaneously provide assistance and express emotions. In future work, more in-depth investigations are needed for determining semantic clarity.

For future works, considering the fact that only one type of soft robotic hand was evaluated in this study, subsequent research could conduct comparative studies involving soft robots with varying degrees of anthropomorphism and diverse product features. Additionally, it is also worth exploring whether users’ safety judgments of the same interactive motions differ based on different expectations in various application scenarios (leaning towards functionality or social interaction). Furthermore, as participants’ self-reported scores on risk tolerance could be biased by various factors, it may also be valuable, in future work, to consider providing participants with fixed priming stimuli (such as news about robotic hands causing harm or helping people) before interaction to initiate and reinforce different attitudes.

## 6. Conclusions

Based on existing work, this study established a conceptual framework for perceived safety and identified applicable evaluation methods. Through user experiments, the following insights were obtained: (1) Key factors influencing user perception of safety: motion speed and proximity are the primary factors users consider when evaluating the safety of interactive motions, followed by contact force, semantics, and size of contact area. (2) Characteristics of motions perceived as safe: simple, slow, easily understandable, predictable, and motions resembling everyday human-to-human contact are more readily accepted by users. (3) Impact of user attitude on perceived safety assessment: Users’ technical acceptance and willingness to tolerate risks concerning the soft robotic hand influence their evaluation of safety. (4) Perceived features of the soft robotic hand affecting safety perception: during interaction, users visually focus on and follow the specific parts of the soft robotic hand involved in contact; thus, the perceived safety of the soft robotic hand could be enhanced by optimizing the shape, color, and texture of critical contact components. Further in-depth research on perceived safety can be conducted with different forms of soft robots, diverse application environments, and varied interaction purposes, to better accommandate diverse user needs in HSRI.

## Figures and Tables

**Table 1:** Detailed information of existing safety assessment scales in HRI.

Authors and Publish Year	Scales Name	Application Fields	Key Safety-Related Factors
Nomura et al., 2006 [51]	Negative Attitude Toward Robot Scale (NARS)	General HRI	Situations, Social Influence and Emotions in Interaction with Robots
Bartneck et al., 2009 [40]	GODSPEED Series Questionnaire	General HRI	Perceived Safety (Anxious/Relaxed; Agitated/Calm; Quiescent/Surprised)
Kamide et al., 2012 [42]	Psychological Scale for Safety of Humanoid	Humanoid rigid robot	Acceptance; Harmlessness; Performance
Carpinella et al., 2017 [53]	Robotic Social Attributes Scale (RoSAS)	General HRI	Discomfort (Scary; Strange; Awkward; Dangerous; Awful; Aggressive)
Akalin et al., 2019 [39]	Questionnaire of Sense of Safety and Security	General HRI	Comfort; Experience; Sense of Security; Trust; Sense of Control

**Table 2:** Significant differences in perceived safety scores among different user groups.

Participant Group	LA	MA	LT	HMT
Low Acceptance (LA)	/	0.000 **	0.000 **	0.039 *
Medium Acceptance (MA)		/	0.001 **	0.000 **
Low Risk Tolerance (LT)			/	0.004 **
High-to-Medium Risk Tolerance (HMT)				/

* *p* < 0.05, ** *p* < 0.01.

**Table 3:** Significant differences in perceived safety scores among different user types.

Participant Type	Proactive	Tech-Savvy	Conservative	Cautious
Proactive (HA/HMT)	/	0.000 **	NS	0.000 **
Tech-savvy (HA/LT)		/	0.000 **	0.000 **
Conservative (MA/MT)			/	0.000 **
Cautious (MA/MT)				/

** *p* < 0.01, NS: not significant.

## Data Availability

Most of the data presented in this study are available in Appendix A and supplementary materials. The raw data of questionnaire results are available on request from the corresponding author. These data are not publicly available due to privacy reasons.

## Supplementary Materials

The following supporting information can be downloaded at: https://www.mdpi.com/article/10.3390/biomimetics9010058/s1, Videos S1–S15.

## Appendix A

**Table A1:** The mechanical features of the 15 interactive motions.

No.	Interactive Motion	Contact Area		Pneumatic Pressure (kPa)	Motion Speed (°/s)	Motion Acceleration (°/s^2)
		Human	Robot			
1	Fast Poking	Forearm	Forefinger	31	119.00	793.38
2	Slow Poking	Forearm	Forefinger	31	14.88	99.17
3	Poking Nearby	No contact	Forefinger	31	74.38	495.86
4	Fast Tapping	Forearm	Four fingers	0	119.00	793.38
5	Slow Tapping	Forearm	Four fingers	0	14.88	99.17
6	Tapping Nearby	No contact	Four fingers	0	89.25	595.03
7	Slow Stroking	Forearm	Four fingers	0	29.75	198.34
8	Fast Stroking	Forearm	Four fingers	0	74.38	495.86
9	Stroking Nearby	No contact	Four fingers	0	44.63	297.52
10	(Slow) Pinching	Forearm	Thumb and forefinger	33	74.38	495.86
11	(Slow) Grasping	Forearm	Five fingers	35	74.38	495.86
12	Grasping Nearby	No contact	Five fingers	35	74.38	495.86
13	Sudden Release	Forearm	Four fingers	11	0.00	0.00
14	Constrain Nearby	non-contact	Four fingers	11	0.00	0.00
15	Shaking Nearby	No contact	Five fingers	0	148.75	991.72

**Table A2:** List of participants and their types.

No.	Sex	Acceptance Score *	Risk Tolerance *	Participant Type
P12	M	4.333/High	4.000/High	“Proactive” High Acceptance; High-to-Moderate Risk Tolerance (HA/HMT)
P14	M	4.167/High	4.333/High	
P16	F	4.167/High	4.333/High	
P26	F	4.167/High	4.333/High	
P35	M	4.167/High	4.000/High	
P10	F	4.833/High	4.333/High	
P2	M	4.000/High	3.000/Moderate	
P1	M	4.167/High	3.000/Moderate	
P13	F	4.833/High	3.000/Moderate	
P7	F	4.333/High	3.333/Moderate	
P21	F	4.167/High	3.667/Moderate	
P15	F	4.833/High	3.667/Moderate	
P18	M	4.167/High	1.000/Low	“Tech-savvy” High Acceptance; Low Risk Tolerance (HA/LT)
P30	F	4.000/High	2.333/Low	
P27	F	4.000/High	2.667/Low	
P9	M	4.167/High	2.667/Low	
P33	F	2.833/Moderate	3.000/Moderate	“Conservative” Moderate Acceptance; Moderate Risk Tolerance (MA/MT)
P5	M	3.000/Moderate	3.000/Moderate	
P17	F	3.000/Moderate	3.333/Moderate	
P3	M	3.667/Moderate	3.333/Moderate	
P4	M	3.833/Moderate	3.333/Moderate	
P28	F	3.833/Moderate	3.667/Moderate	
P23	F	3.500/Moderate	1.667/Low	“Cautious” Moderate Acceptance; Low Risk Tolerance (MA/LT)
P31	M	3.500/Moderate	2.000/Low	
P19	F	3.333/Moderate	2.333/Low	
P29	F	3.333/Moderate	2.333/Low	
P22	F	3.667/Moderate	2.333/Low	
P25	F	3.667/Moderate	2.333/Low	
P32	F	3.667/Moderate	2.333/Low	
P24	M	3.167/Moderate	2.667/Low	

* The maximum score for these two indicators is 5 points.

**Table A3:** Perceived safety assessment questionnaire used in the user experiments.

No.	Item	Scale	Description
1	How would you rate your acceptance of the interaction between the soft robotic hand and the human user in the video?	5-point scale	Not Accepting -Accepting
2	Do you consider the motion of the soft robotic hand in the video to be trustworthy?	5-point scale	Not Trustworthy -Trustworthy
3	Does the motion of the soft robotic hand in the video make you feel relaxed or anxious?	5-point scale	Relaxed-Anxious
4	Do you think the motion of the soft robotic hand in the video is fluent?	5-point scale	Not Fluent-Fluent
5	Do you believe the motion of the soft robotic hand in the video is secure?	5-point scale	Unsecure-Secure
6	Do you believe the soft robotic hand in the video is functioning normally or malfunctioning?	5-point scale	Malfunctioning -Functioning Normally
7	Overall, do you consider this soft robotic hand to be safe?	5-point scale	Unsafe-Safe
8	Would you like to personally try using this soft robotic hand and interact with it as shown in the video?	5-point scale	Unwilling -Willing
9	What do you think is the most likely meaning of the motion performed by the soft robotic hand in the video?	Single-choice question	Prompt/alert; Emotion expression; Assistance provision; Malfunction; and Other

**Table A4:** Significant differences in perceived safety scores among different interactive motions.

Motion Name	Slow Poking	Slow Tapping	Slow Stroking	(Slow) Pinching	(Slow) Grasping	Fast Poking	Fast Tapping	Fast Stroking	Sudden Release	Poking Nearby	Tapping Nearby	Stroking Nearby	Grasping Nearby	Constrain Nearby	Shaking Nearby
Slow Poking	/	NS	NS	NS	0.000 **	0.000 **	0.000 **	0.030 *	0.000 **	0.000 **	0.000 **	0.011 *	0.000 **	0.000 **	0.000 **
Slow Tapping		/	NS	0.000 **	0.000 **	0.000 **	0.000 **	0.002 **	0.000 **	0.000 **	0.000 **	0.001 **	0.000 **	0.000 **	0.000 **
Slow Stroking			/	0.046 *	0.000 **	0.003 **	0.000 **	0.026 *	0.000 **	0.001 **	0.000 **	0.019 *	0.000 **	0.000 *	0.000 *
(Slow) Pinching				/	0.001 **	NS	0.000 **	NS	0.000 **	0.018 *	0.001 **	NS	0.003 **	0.000 **	0.000 **
(Slow) Grasping					/	NS	NS	0.010 *	NS	NS	NS	NS	NS	NS	NS
Fast Poking						/	0.046 *	NS	0.002 **	NS	NS	NS	NS	0.017 *	0.042 *
Fast Tapping							/	0.009 **	NS	NS	NS	0.015 *	NS	NS	NS
Fast Stroking								/	0.004 **	NS	NS	NS	NS	0.008 **	0.005 **
Sudden Release									/	0.038 *	NS	0.007 **	0.046 *	NS	NS
Poking Nearby										/	NS	NS	NS	0.016 *	0.024 *
Tapping Nearby											/	0.004 **	NS	NS	NS
Stroking Nearby												/	0.043 *	0.000 *	0.002 **
Grasping Nearby													/	0.037 *	NS
Constrain Nearby														/	NS
Shaking Nearby															/

* *p* < 0.05, ** *p* < 0.01, NS: not significant.
